# The Efficacy and Underlying Mechanism of Sulfone Derivatives Containing 1,3,4-oxadiazole on Citrus Canker

**DOI:** 10.3390/molecules200814103

**Published:** 2015-08-04

**Authors:** Pei Li, Yuhua Ma, Junliang Zhou, Hui Luo, Jiawen Yan, Yongya Mao, Zhuang Wang

**Affiliations:** 1 State Key Laboratory Breeding Base of Green Pesticide and Agricultural Bioengineering/Key Laboratory of Green Pesticide and Agricultural Bioengineering, Ministry of Education, Guizhou University, Guiyang 550025, China; E-Mail: luohui8732@163.com; 2 Guizhou Fruit Institute, Guizhou Academy of Agricultural Sciences, Guiyang 550025, China; E-Mails: gsszjl2008@163.com (J.L.Z.); yanjw200709@126.com (J.W.Y.); mao3354@126.com (Y.Y.M.); guhun_145@163.com (Z.W.)

**Keywords:** sulfone derivatives, *Xanthomonas citri* subsp. *citri*, citrus canker, antibacterial bioassay, underlying mechanism

## Abstract

The objectives of the current study were to isolate and identify the pathogen responsible for citrus canker and investigate the efficacy of sulfone derivatives containing 1,3,4-oxadiazole moiety on controlling citrus canker caused by *Xanthomonas citri* subsp. *citri* (*Xcc*) under *in vitro* and field conditions. In an *in vitro* study, we tested eight sulfone derivatives against *Xcc* and the results demonstrated that compound **3** exhibited the best antibacterial activity against *Xcc*, with a half-maximal effective concentration (EC_50_) value of 1.23 μg/mL, which was even better than those of commercial bactericides Kocide 3000 (58.21 μg/mL) and Thiodiazole copper (77.04 μg/mL), respectively. Meanwhile, under field experiments, compound **3** treatments demonstrated the highest ability to reduce the disease of citrus canker in leaves and fruits in two different places relative to an untreated control as well as the commercial bactericides Kocide 3000 and Thiodiazole copper. Meanwhile, compound **3** could stimulate the increase in peroxidase (POD), polyphenol oxidase (PPO), and phenylalanine ammonia lyase (PAL) activities in the navel orange leaves, causing marked enhancement of plant resistance against citrus canker. Moreover, compound **3** could damage the cell membranes, destruct the biofilm formation, inhibit the production of extracellular polysaccharide (EPS), and affect the cell membrane permeability to restrain the growth of the bacteria.

## 1. Introduction

Citrus canker, a serious disease of most commercial citrus cultivars in subtropical citrus-producing areas of the world, has a significant impact on national and international citrus markets and trade [1,2,3]. Citrus canker is a disease that is characterized by the formation of necrotic raised lesions on leaves, stems, and fruit of citrus trees, including limes, oranges, and grapefruit. Once infected with the disease, citrus canker can cause defoliation, twig dieback, general tree decline, blemished fruit, and premature fruit drop [4]. Citrus canker is caused by the bacterial pathogen *Xanthomonas citri* subsp. *citri* (*Xcc*) [5,6,7]. This bacterium is dispersed in rain splash often associated with wind [8,9,10,11,12] and enters the plant directly through stomata or through wounds, and then it grows in the intercellular spaces of the spongy mesophyll [1]. At present, copper-containing bactericides are the primary control measure for citrus canker. However, long-term use of copper bactericides not only led to resistance to copper in xanthomonad populations but also affected the environment and plant health [13]. Therefore, searching for new antibacterial agents for controlling the disease remains a daunting task in pesticide science.

**Figure 1:**
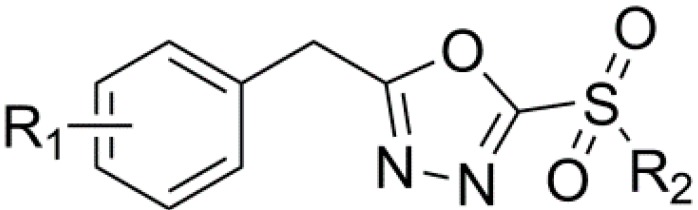
Compounds previously reported against rice bacterial leaf blight and leaf streak.

Over the past few years, we have attracted considerable attention on the studies of the synthesis and bioactivity of sulfone derivatives containing 1,3,4-oxadiazole moiety and demonstrated that sulfone derivatives containing 1,3,4-oxadiazole moiety (Figure 1) display potent antibacterial activities against rice bacterial leaf blight and leaf streak. Specifically, compound 2-(methyl sulfonyl)-5-(4-fluorobenzyl)-1,3,4-oxadiazole (CAS Registry Number: 1596304-56-1) showed the best antibacterial activity against rice bacterial leaf blight and leaf streak caused by *Xanthomonas oryzae* pv. *oryzae* (*Xoo*) and *Xanthomonas oryzae* pv. *oryzicola* (*Xoc*), with the half-maximal effective concentration (EC_50_) values of 1.07 and 7.14 μg/mL, respectively [14]. However, in our previous work, we only reported and discussed the compound activities in the control of rice bacterial leaf blight and leaf streak. The biological effects and the underlying mechanism of these sulfone derivatives containing 1,3,4-oxadiazole moiety on citrus canker were not reported.

The objectives of the current study were to isolate and identify the pathogen responsible for citrus canker and investigate the efficacy and the underlying mechanism of sulfone derivatives containing 1,3,4-oxadiazole moiety on controlling citrus canker caused by *Xcc* under *in vitro* and field conditions. To the best of our knowledge, this is the first report on the bioactivity evaluation and the underlying mechanism of sulfone derivatives containing 1,3,4-oxadiazole moiety on citrus canker.

## 2. Results and Discussion

### 2.1. DNA Extraction, PCR Amplification, Sequencing, and Identification of Species

The genomic DNA was collected using the TIANamp bacteria DNA distilling kit (Tiangen-Biotech Corporation LTD, Beijing, China) and the DNA concentration and quality, estimated using an ASP-3700 Spectrophotometer (ACTGene, Piscataway, NJ, USA), were 125.5 ng/μL and 1.82 (OD_260_/OD_280_), respectively.

**Figure 2:**
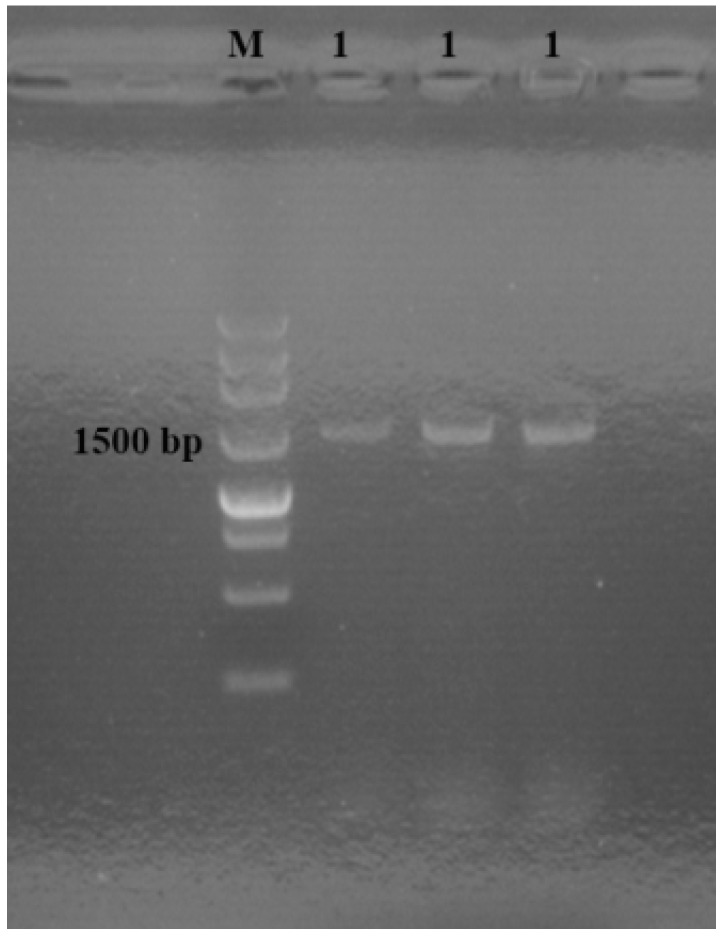
PCR analysis of the genomic DNA. M: 250 bp DNA Ladder marker; 1: PCR amplicons.

Then, the genomic DNA was conducted to PCR amplification using the bacterial universal primer pair 27F/1492R. After PCR analysis, the whole PCR reaction volume was electrophoresed for 25 min onto 1.5% agarose gel in Tris-acetate-EDTA (TAE) buffer with 5 μL 4S green nucleic acid stain. As shown in Figure 2, the PCR amplicon with a molecular weight of about 1500 bp was obtained. Then, the whole 16S rDNA sequence of the sample, sequenced at Sangon Corporation (Shanghai, China), showed that the sequence identity between the sample and *Xanthomonas citri* subsp. *citri* (accession number: CP008989) was 99%. Thus, it appears to be likely that the strain is *Xanthomonas citri* subsp. *citri*.

### 2.2. In Vitro Antibacterial Bioassay

In this study, the inhibitory effect of the target compounds **1**–**8** were evaluated for their antibacterial activities *in vitro* against *Xcc* via the turbidimeter test. For comparison, the activity of the commercial bactericides Kocide 3000 and Thiodiazole copper, two positive controls, were evaluated in the same conditions. The results of the preliminary bioassays, as listed in Table 1, indicated that all of the title compounds demonstrated good antibacterial bioactivities against *Xcc*. Table 1 showed that the EC_50_ values of compounds **1**–**8** against *Xcc in vitro* were 6.52, 27.80, 1.23, 16.16, 12.28, 48.54, 2.47, and 30.10 μg/mL, respectively, which were even better than those of Kocide 3000 (58.21 μg/mL) and Thiodiazole copper (77.04 μg/mL). Especially, compound **3** demonstrated the excellent inhibitory effect against *Xcc* with EC_50_ value of 1.23 μg/mL.

**Table 1:** The inhibitory effect of the title compounds against *Xcc*.

No.	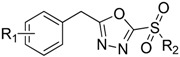		Toxic Regression Equation	*r*	EC_50_ (μg/mL)
	R_1_	R_2_			
**1**	H	–CH_3_	y = 1.42x + 3.84	0.99	6.52 ± 1.19
**2**	H	–CH_2_CH_3_	y = 1.56x + 2.75	0.98	27.80 ± 2.76
**3**	4-F	–CH_3_	y = 1.56x + 4.86	0.99	1.23 ± 0.97
**4**	4-F	–CH_2_CH_3_	y = 1.42x + 3.28	0.99	16.16 ± 2.21
**5**	4-Cl	–CH_3_	y = 1.56x + 3.30	0.99	12.28 ± 1.76
**6**	4-Cl	–CH_2_CH_3_	y = 1.53x + 2.43	0.99	48.54 ± 2.78
**7**	2,4-2Cl	–CH_3_	y = 1.58x + 4.38	0.96	2.47 ± 0.69
**8**	2,4-2Cl	–CH_2_CH_3_	y = 1.40x + 2.92	0.97	30.10 ± 3.87
Kocide 3000			y = 1.61x + 2.15	0.98	58.21 ± 2.77
Thiodiazole copper			y = 2.15x + 0.94	0.98	77.04 ± 1.96

### 2.3. Field Trials against Citrus Canker

Field trials of compound **3** against citrus canker were conducted in two different places, Congjiang and Luodian, Guizhou Province, in May 2014. Results were summarized in Table 2. Table 2 indicated that, 14 days after the third spraying in Congjiang, Guizhou Province, the control efficiencies in leaves and fruits of compound **3** against citrus canker were 66.31% and 69.03%, respectively, which were even better than those of Kocide 3000 (55.13% and 53.76%, respectively) and Thiodiazole copper (61.47% and 57.73%, respectively). Meanwhile, the result, shown in Table 2, indicated that, 14 days after the third spraying in Luodian, Guizhou Province, the control efficiencies in leaves and fruits of compound **3** against citrus canker, with the values of 60.43% and 64.51%, respectively, were also superior to those of Kocide 3000 (50.93% and 52.77%, respectively) and Thiodiazole copper (55.72% and 56.52%, respectively).

**Table 2:** Control efficiency (mean value ± SD) of the testing compound against citrus canker in the field trials.

Treatment	Congjiang, Guizhou Province		Luodian, Guizhou Province	
	In Leaves ^a^	In Fruits ^a^	In Leaves ^a^	In Fruits ^a^
**3**	66.31 ± 2.45A	69.03 ± 5.12A	60.43 ± 5.67A	64.51 ± 2.23A
Kocide 3000	55.13 ± 5.63B	53.76 ± 4.43B	50.93 ± 4.34C	52.77 ± 5.98B
Thiodiazole copper	61.47 ± 2.32C	57.73 ± 3.73C	55.72 ± 2.86B	56.52 ± 3.76C

^a^: The statistical analysis was conducted by the ANOVA method at the condition of equal variances assumed (*p* > 0.05) and equal variances not assumed (*p* < 0.05). Different letters indicate the values of control efficiency with significant difference among different treatment groups at *p* < 0.05.

### 2.4. Determination of Peroxidase (POD), Polyphenol oxidase (PPO), and Phenylalanine ammonia lyase (PAL) Activities

**Figure 3:**
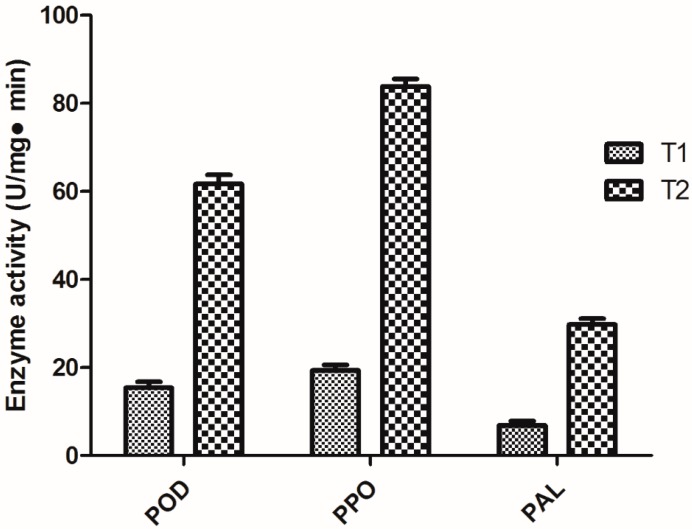
The changes of peroxidase (POD), polyphenol oxidase (PPO), and phenylalanine ammonia lyase (PAL) activities (mean value ± SD). T1: Untreated blank control; T2: Treatment group.

As shown in Figure 3, seven days after the third spraying, the POD, PPO, and PAL activities of the navel orange leaves, treated with compound **3**, were 61.66, 83.80, and 29.73 U/mg·min, respectively. Meanwhile, the POD, PPO, and PAL activities of the untreated blank control were 15.36, 19.30, and 6.86 U/mg·min, respectively. Obviously, Figure 3 showed that, compared with the untreated blank control, the POD, PPO, and PAL activities in the treatment group increased by approximately 4.01, 4.34, and 4.33 times, respectively. In conclusion, the enzyme activities changes of POD, PPO, and PAL preliminarily demonstrated that compound **3** can improve the disease resistance of plants that rely on inducible defense responses in the form of enzymes that are activated for controlling citrus canker.

### 2.5. Effect on the Integrity of Bacterial Cell Membranes

**Figure 4:**
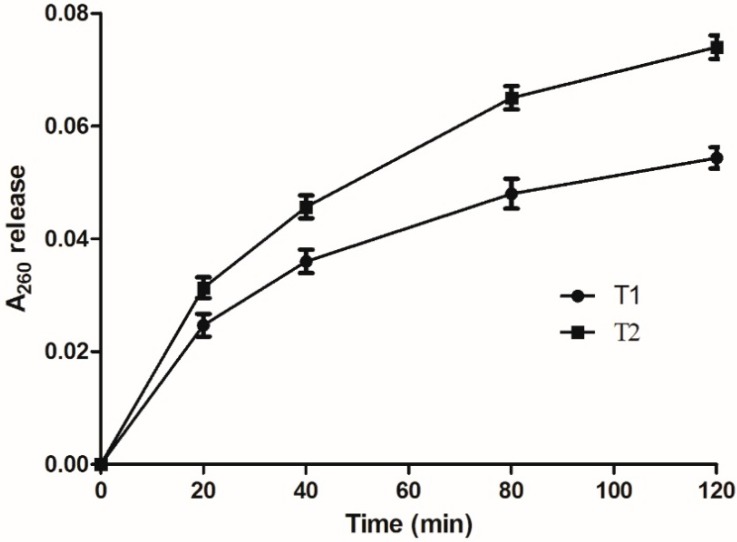
Release of cell membranes absorbing at 260 nm for *Xcc* treated with compound **3**. T1: 10 μg/mL; T2: 20 μg/mL.

The release of intracellular components that absorb at 260 nm is an indication of membrane damage. As shown in Figure 4, when bacterial suspensions were treated with different concentrations of compound **3**, the A_260_ increased rapidly at first, then slowed its rate up to 120 min. A_260_ values were greater in suspensions treated with 20 μg/mL than with 10 μg/mL of compound **3**. Thus, the damage of cell membranes by compound **3** is concentration-dependent, which agrees with the findings for bactericidal activity.

### 2.6. Effect on the Biofilm Formation

**Figure 5:**
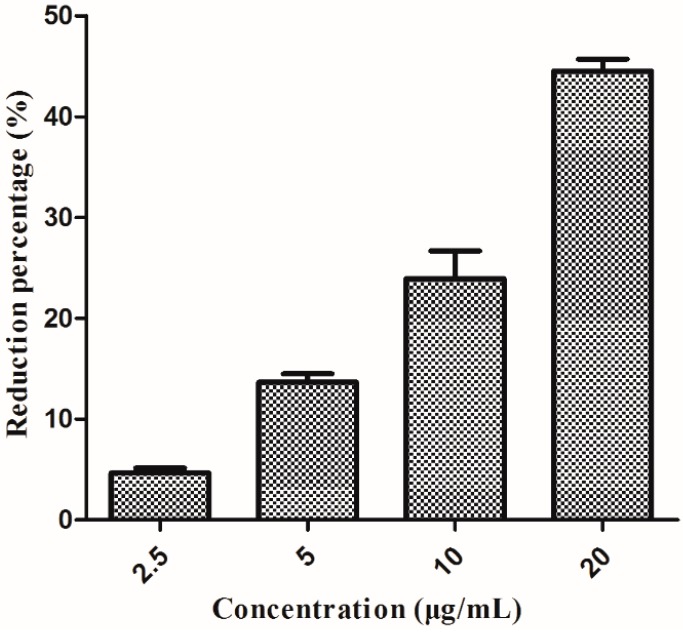
Effect (mean value ± SD) of compound **3** on biofilm formation of *Xcc*.

To study the effect of compound **3** on biofilm formation, compound **3** was hypothesized to be involved in biofilm formation. As shown in Figure 5, compound **3** could significantly affect the biofilm formation with the reduction percentages of 4.67%, 13.65%, 26.54%, and 43.32% at the concentration of 2.5, 5, 10, and 20 μg/mL, respectively. The results revealed that the destruction of biofilm formation may play an important role in the antibacterial activity of compound **3** against *Xcc*.

### 2.7. Effect on Cell Membrane Permeability of Xcc

**Figure 6:**
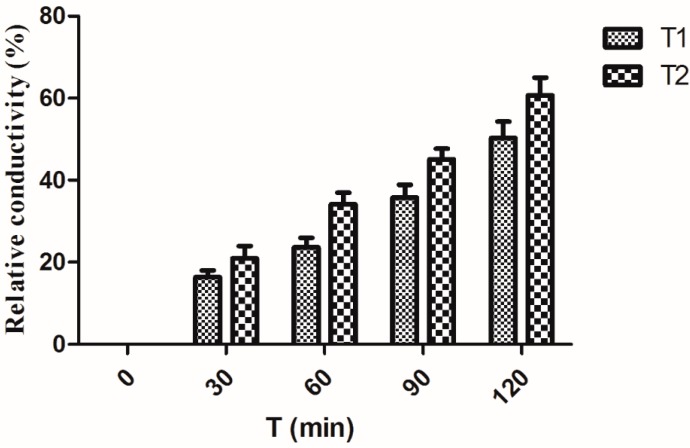
Electric conductivity (mean value ± SD) of cell suspensions for *Xcc* treated with compound **3**. T1: 10 μg/mL, T2: 20 μg/mL.

We determined the electric conductivity of the cell suspensions for *Xcc* treated with compound **3** at the ultimate concentrations of 10 and 20 μg/mL, respectively. As shown in Figure 6, the electric conductivity showed a time-dependent increasing manner. It was found that the electric conductivity of suspensions for *Xcc* began to increase after being treated with compound **3**, with the concentration of 10 and 20 μg/mL, respectively, and when the incubation time was 120 min, the relative conductivity increased by 50.28% and 60.69% for *Xcc* compared with the untreated blank control.

### 2.8. Determination of Exopolysaccharide (EPS) Content

**Figure 7:**
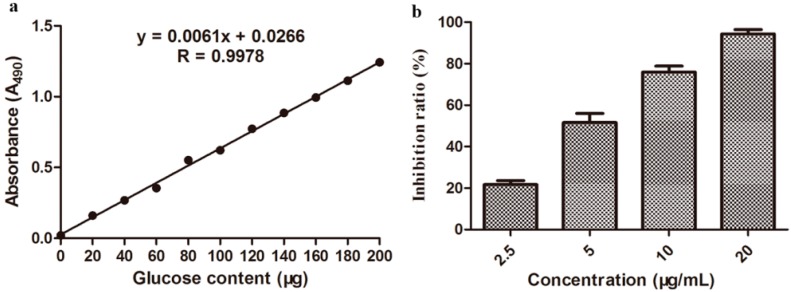
Standard curve for determination of EPS content (**a**) and inhibition rates (mean value ± SD) of EPS of *Xcc* with different concentrations (2.5, 5, 10, and 20 μg/mL) of compound **3** treatment (**b**).

The EPS content was determined by comparison of absorbance at 490 nm of inoculated *Xcc* either treated or untreated with compound **3**. EPS content was calculated using the standard curves (Figure 7a). As shown in Figure 7b, compound **3**, at the concentrations of 2.5, 5, 10, and 20 μg/mL, could obviously inhibit the EPS production of *Xcc*, with inhibition rates of 21.69%, 51.60%, 75.95%, and 94.34%, respectively. These results demonstrated that compound **3** could reduce EPS production to lower the pathogenic ability of *Xcc*.

## 3. Experimental Section

### 3.1. Bacteria Isolation and Purification

Bacteria were isolated from infected leaves and fruits of navel oranges collected from Congjiang, Guizhou Province of China using the previously described method [15] with some modifications. Small portions of infected fruits and leaves were disinfected using 75% ethanol, and then washed three times with sterile distilled water. These tissue portions were transferred to Nutrient Agar (NA) plates and incubated at 28–30 °C for approximately 48 h. Bacteria colonies growing around the tissue mass were aseptically moved using an inoculation loop and transferred to flesh NA plates and incubated at 28–30 °C for approximately 48 h. Discrete bacterial colonies were selected and re-streaked on flesh NA plates. Individual colonies were isolated, sub-cultured twice to ensure purity, and the single-spore isolates were stored in sterile distilled water at 4 °C for later use.

### 3.2. DNA Extraction, PCR Amplification, and Sequencing of Species

Prior to DNA extraction, each isolate was sub-cultured on Nutrient Broth (NB) medium at 28–30 °C for 48 h. Approximately 25 mg of bacteria were collected for genomic DNA extraction using the TIANamp bacteria DNA distilling kit (Tiangen-Biotech Corporation LTD, Beijing, China) and DNA concentration and quality were estimated using an ASP-3700 spectrophotometer (ACTGene, Piscataway, NJ, USA).

The sequence of the 16S rDNA of the sample was obtained from the total of the bacteria by PCR amplification with the bacterial universal primer pair 27F/1492R [16], which consisted of a forward primer 27F (5′-AGAGTTTGATCCTGGCTCAG-3′) and a reverse primer 1492R (5′-TACGGCTACCTTGTTACGACTT-3′). Reactions were conducted in a final volume of 20 μL, which contained 10 μL of Premix Taq Ver., 2.0 μL of plus dye (Takara, Dalian, China), 7.2 μL of sterile distilled water, 0.4 μL of each primer, and 2 μL of genomic DNA. The PCR amplification conditions in the thermocycler were set as follows: 5 min at 95 °C followed by 30 cycles at 95 °C for 30 s, 1 min at 55 °C and 90 s at 72 °C with a final extension of 5 min at 72 °C. After PCR analysis, the whole PCR reaction product was electrophoresed for 25 min onto 1.5% agarose gel with 5 μL 4S green nucleic acid stain in Tris-acetate-EDTA (TAE) buffer. Then, the amplicons were sequenced at Sangon Corporation (Shanghai, China). The DNA sequences of the isolates were used to search for sequence similarity against the National Center for Biotechnology Information (NCBI) database using the Standard Nucleotide BLAST program. 

### 3.3. In Vitro Antibacterial Bioassay

In this study, eight of the title compounds were evaluated for their antibacterial activities against *Xcc*, isolated from the infected fruits of navel oranges, by the turbidimeter test [17] *in vitro*. Dimethylsulfoxide in sterile distilled water served as a blank control and Kocide 3000 and Thiodiazole copper, commonly used as the principal tools for controlling citrus canker in China at present, served as two positive controls. Approximately 40 μL of Nutrient Broth (NB) medium containing *Xcc*, incubated on the phase of logarithmic growth, was added to 5 mL of NB medium containing the test compounds or the commercial bactericides Kocide 3000 and Thiodiazole copper. The inoculated test tubes were incubated at 28–30 °C and continuously shaken at 180 rpm for 24–48 h until the bacteria were incubated on the phase of logarithmic growth. The growth of the cultures was monitored on a Model 680 microplate reader (BIO-RAD, Hercules, CA, USA) by measuring the optical density at 595 nm (OD_595_) and then the inhibition rate *I* was calculated by the following formula:

(1) $$\text{Inhibition rate}\;I\left(\text{\%}\right)=\frac{C\;-\;T}{C}\times 100$$

where *C* is the corrected turbidity value of bacterial growth on untreated NB (blank control), and *T* is the corrected turbidity value of bacterial growth on treated NB, and *I* represents the inhibition rate.

On the basis of previous bioassays, the results of antibacterial activities (expressed by EC_50_) of the title compounds against *Xcc* were also evaluated and calculated with SPSS 17.0 software. The experiment was repeated three times.

### 3.4. Field Trial against Citrus Canker

In order to further determine the activities of the title compounds, which showed better antibacterial activities against *Xcc in vitro*, field trials of compound **3** against citrus canker were conducted in Congjiang and Luodian, Guizhou Province, respectively, in 2014. The citrus variety is navel orange and the effect of the natural infection of *Xcc* was studied in a field having suffered citrus canker for several years. Sterile distilled water served as a blank control, whereas the commercial bactericides Kocide 3000 and Thiodiazole copper served two positive controls. The solutions of compound **3** (20% suspension concentrate (SC), 500 fold dilution, 300 g ai/ha) and the commercial bactericides Kocide 3000 (46% water dispersible granule (WDG), 1000 fold dilution, 300 g ai/ha) and Thiodiazole copper (20% SC, 500 fold dilution, 300 g ai/ha) were sprayed three times on the foliage once every seven days. Approximately 1.5 L per tree of spray, depending on the size of the testing trees, was applied with a backpack sprayer (Model 3WBS-16C, Shun Industrial Co., Ltd, Taizhou, China). The experimental design area of the plot was about 20 m^2^ with five trees, three replicates were conducted. The disease incidence was investigated on the 14th day after the third spraying and the control efficiencies in leaves and fruits were calculated by the following formula:

(2) $$\text{Control efficiency}\;I\left(\text{\%}\right)=\frac{CK\;-\;PT}{CK\;}\times 100$$

where *CK* represents the disease incidence of the untreated plot, *PT* represents the disease incidence of the treatment plot, and *I* represents the control efficiency.

### 3.5. Determination of POD, PPO, and PAL Activities

Disease resistance in plants is associated with the activation of a wide array of defense responses that slow down or halt infection at certain stages of the host-pathogen interaction. The defense mechanisms include preexisting physical and chemical barriers that interfere with pathogen establishment. Other methods of protection rely on inducible defense responses in the form of enzymes that are activated upon infection [18] or plant activators [19,20,21,22,23]. The interaction between the pathogen or plant activators and the host plant induces some changes primarily in the activity of enzymes, particularly PAL, POD, PPO, *etc.* [24,25,26]. PAL is the primary enzyme in the phenylpropanoid pathway, which leads to the conversion of l-phenylalanine to trans-cinnamic acid with the elimination of ammonia, and it is the key enzyme in the synthesis of several defense-related secondary compounds such as phenols and lignin [27]. Meanwhile, PPO is a nuclear-encoded enzyme that catalyzes the oxygen-dependent oxidation of phenols to quinones, and PPO levels in a plant increase when a plant is wounded or infected [18]. Moreover, POD constitutes a class of enzymes extensively distributed in plants and it has been shown that POD plays an active role in metabolism and has been suggested as a defense response of plants to stress [28].

In view of the above findings and as an extension of our studies on the further research of whether the testing compound can improve the disease resistance of plants that rely on inducible defense responses in the form of enzymes that are activated, the enzymatic activities of PPO, POD, and PAL were determined. Seven days after the third spraying of compound **3**, the leaves were collected and powdered by liquid nitrogen and the leaves that were untreated were used as a blank control. Powdered samples of leaves (0.5 g) were homogenized with cold extraction buffer containing 20 mL of 0.01 M sodium phosphate buffer (PBS, pH 5.9) for the assay of the enzymatic activities of PPO and POD. PAL activity was measured in powders extracted with 0.01 M PBS (pH 8.8) containing 5 mM β-mercaptoethanol and 5% polyvinylpyrrolidone (PVP). The extracts were filtered through two layers of miracloth and the filtrates were centrifuged at 12,000 rpm at 4 °C for 15 min.

PPO and POD activities were determined according to the reported methods [29,30]. For PPO analysis, 1 mL of supernatant was mixed with 3 mL of PBS (0.01 M, pH 5.9) and 1 mL pyrocatechol (0.2 M). A control was similarly prepared by adding 1 mL of PBS instead of 1 mL of protein extraction. Change in absorbance at 410 nm was measured by spectrophotometer. One unit of PPO activity was defined as a change of one in absorbance per minute [31].

(3) $$\text{PPO activity}=\frac{\text{Δ}A_{410}\;\times \;V_{T}}{W\;\times \;Vs\;\times \;0.01\;\times \;t\;}\;\left[\text{U}/\text{mg}\cdot \text{min}\right]$$

POD activity was measured in a reaction mixture consisting of 0.1 mL of supernatant, 0.4 mL of 0.05 M guaiacol, and 3.5 mL of 0.01 M PBS (pH 5.9). The increase in absorbance at 470 nm was measured by spectrophotometer after 1 mL H_2_O_2_ was added. A control was similarly prepared by adding 1 mL of PBS instead of 1 mL of H_2_O_2_. One unit of enzyme activity was defined as a change of one in absorbance per minute. POD activity was calculated as follows:

(4) $$\text{POD activity}=\frac{\text{Δ}A_{470}\;\times \;V_{T}}{W\;\times \;Vs\;\times \;0.1\;\times \;t}\;\left[\text{U}/\text{mg}\cdot \text{min}\right]$$

PAL activity was assayed according to Assis *et al.* [32] with slight modifications. First, 1 mL of supernatant was mixed with 2 mL of 50 mM sodium borate buffer (BBS, pH 8.8) and 1 mL of 20 mM l-phenylalanine and incubated in a water bath at 40 °C for 30 min. Then the reaction was stopped by adding 1 mL of 1 M hydrochloric acid (HCl). A control was similarly prepared by adding 1 mL of BBS (pH 8.8) instead of 1 mL of protein extract. PAL activity was assayed by spectrophotometer at 290 nm. One unit of enzyme activity was defined as the increase of one in absorbance per hour. PAL activity was calculated as follows:

(5) $$\text{PAL activity}=\frac{\text{Δ}A_{290}\;\times \;V_{T}}{W\;\times \;Vs\;\times \;0.01\;\times \;t\;}\;\left[\text{U}/\text{mg}\cdot \text{min}\right]$$

where *W* (mg) is the total protein content of the leaves, *t* (min) is the reaction time, *V_T_* (mL) is the total volume of protein extract, and *V_s_* (mL) is the amount of protein extract used for detection.

### 3.6. Effect on the Cell Membrane Integrity

Bacterial cell membrane integrity was examined by determination of the release of material absorbing at 260 nm following the reported method [33] with slight modifications. Bacterial cultures, in the mid-exponential growth phase, were harvested, washed, and re-suspended in 0.75% NaCl solution. The final cell suspension was adjusted to an absorbance at 595 nm (OD_595_) of 0.6. A 0.25 mL portion of compound **3** was mixed with 0.25 mL of bacterial cell suspension to give the final concentration of 10 and 20 μg/mL, respectively, and the release over time of materials absorbing at 260 nm was monitored with a Perkin-Elmer model 554 UV-Vis recording spectrophotometer.

### 3.7. Effect on the Biofilm Formation

It is reported that biofilm formation plays an important role in early infection of *Xcc* on host leaves [13]. In this study, the effect on the biofilm formation was studied in 96-well plates based on the method described previously [34,35] with some modifications. Compound **3**, at the final concentration of 2.5, 5, 10, and 20 μg/mL, respectively, was added into the mid-exponential growth phase bacterial suspension, while the same volume of sterile distilled water was added to the untreated blank controls. The mixture was incubated at 28 °C for 36 h. Following that, 200 μL of the suspension was added to individual wells of 96-well plates and incubated at 28 °C for 12 h without shaking. Each treatment consists of three wells. Then the wells were washed three times with sterile distilled water to remove non-adhered bacteria and the remaining attached bacteria were dried and stained with 200 μL of 0.1% (*w*/*v*) crystal violet for 15 min. The wells were washed to remove non-adsorbed crystal violet solution and immediately solubilized with 200 μL of 33% acetic acid. The solution was monitored on a Model 680 microplate reader (BIO-RAD) by measuring the optical density at 595 nm (OD_595_).

### 3.8. Effect on the Cell Membrane Permeability

To determine the effect of compound **3** on cell membrane permeability of *Xcc*, an isolated single colony of *Xcc* was sub-cultured in 250 mL flasks containing 100 mL of NB medium. The flasks were placed on a rotary shaker at 180 rpm at 28 °C. After 36 h, partial flasks were amended with compound **3** at the ultimate concentration of 10 and 20 μg/mL. The flasks were shaken for an additional 36 h, the cells were collected by centrifugation at 12,000 rpm for 10 min and washed twice with 0.75% NaCl solution. After centrifugation for 10 min, approximately 25 mg of bacteria was suspended in 1 mL of 0.75% NaCl solution. After 0, 30, 60, 90, and 120 min, the electrical conductivity of the 0.75% NaCl solution was measured with a conductivity meter (CON510 Eutech Ltd., Oaklon, Singapore) to assess the extent of leaching of cell contents through cell membranes. After 180 min, the bacteria were boiled for 5 min, and final conductivity was measured. Each experiment was repeated three times. The relative conductivity of cells was calculated as:

(6) $$\text{Relative conductivity}\left(\text{\%}\right)=\frac{\mathrm{Conductivity}}{\mathrm{Final}\;\mathrm{conductivity}}\times 100$$

### 3.9. EPS Content

The quantity of EPS produced by *Xcc* was determined by the phenol-sulfuric acid method [36,37,38] with some modifications. For preparation of an EPS standard curve, 2 mL of a glucose solution (0, 20, 40, 60, 80, 100, 120, 140, 160, 180, and 200 μg of glucose/mL of double-distilled water) and 1 mL of a 5% phenol solution were added to the test tube, respectively, and mixed with a vortex mixer. Then 5 mL of concentrated H_2_SO_4_ was added slowly to the test tubes. The test tubes were then closed with rubber plugs, mixed with a vortex mixer for 10 s, and then incubated for 30 min at 25 °C. The solution absorbance was measured using a Model 680 microplate reader (BIO-RAD) by measuring the optical density at 490 nm (OD_490_). In this case, the greater the absorbance, the higher the glucose concentration. A standard curve was generated by plotting absorbance against glucose concentration.

For the determination of EPS content, an isolated single colony of *Xcc* was sub-cultured in 250 mL flasks containing 100 mL of NB medium at 28 °C with continuous shaking at 180 rpm for 36 h. Then, the partial flasks were supplemented with compound **3** at the ultimate concentration of 2.5, 5, 10, and 20 μg/mL, respectively. The flasks were shaken continuously for an additional 36 h. Then, the flask contents were centrifuged at 12,000 rpm for 10 min, and the supernatants were collected. EPS was precipitated from 1 mL of each supernatant with three volumes of absolute ethanol, collected via centrifugation and dried. Finally, the EPS were dissolved in 10 mL of distilled water, the optical density was measured at 490 nm (OD_490_) using a Model 680 microplate reader (BIO-RAD), and the standard curve was quantified. Sterile-distilled water was used as an untreated blank control. There were three replications for each treatment.

## 4. Conclusions

In this study, the pathogenic bacterium *Xcc*, the cause of citrus canker, was isolated from an infected corm of navel orange fruit and the species was identified via PCR analysis and the amplicons were sequenced. We investigated the efficacy of eight sulfone derivatives containing 1,3,4-oxadiazole moiety on controlling citrus canker caused by *Xcc* under *in vitro* and field conditions. Antibacterial bioassay results indicated that compound **3** demonstrated appreciable control efficiencies against citrus canker under *in vitro* and field conditions, which were even better than those of Kocide 3000 and Thiodiazole copper. Meanwhile, the changes of the enzyme activity of POD, PPO, and PAL in navel orange leaves demonstrated that compound **3** could improve the disease resistance of plants that rely on inducible defense responses in the form of enzymes that are activated for controlling citrus canker. Moreover, compound **3** could damage the cell membranes, destruct the biofilm formation, inhibit the production of EPS, and affect the cell membrane permeability to restrain the growth of the bacteria. This work demonstrated that sulfone derivatives containing 1,3,4-oxadiazole moiety can be used to develop potential bactericides for controlling citrus canker.
